# Evidence for the Nucleo-Apical Shuttling of a Beta-Catenin Like *Plasmodium falciparum* Armadillo Repeat Containing Protein

**DOI:** 10.1371/journal.pone.0148446

**Published:** 2016-02-01

**Authors:** Pallabi Mitra, Enna Dogra Gupta, Tajali Sahar, Alok K. Pandey, Poonam Dangi, K. Sony Reddy, Virander Singh Chauhan, Deepak Gaur

**Affiliations:** 1 Malaria Group, International Centre for Genetic Engineering and Biotechnology (ICGEB), New Delhi, India; 2 Laboratory of Malaria and Vaccine Research, School of Biotechnology, Jawaharlal Nehru University, New Delhi, India; Institut national de la santé et de la recherche médicale - Institut Cochin, FRANCE

## Abstract

Eukaryotic Armadillo (ARM) repeat proteins are multifaceted with prominent roles in cell-cell adhesion, cytoskeletal regulation and intracellular signaling among many others. One such ARM repeat containing protein, ARM Repeats Only (ARO), has recently been demonstrated in both *Toxoplasma* (TgARO) and *Plasmodium* (PfARO) parasites to be targeted to the rhoptries during the late asexual stages. TgARO has been implicated to play an important role in rhoptry positioning i.e. directing the rhoptry towards the apical end of the parasite. Here, we report for the first time that PfARO exhibits a DNA binding property and a dynamic sub-cellular localization between the nucleus (early schizont) and rhoptry (late schizont) during the different stages of the asexual blood-stage life cycle. PfARO possesses a putative nuclear export signal (NES) and the nucleo-apical shuttling was sensitive to Leptomycin B (LMB) suggesting that the nuclear export was mediated by CRM1. Importantly, PfARO specifically bound an A-T rich DNA sequence of the *P*. *falciparum Gyrase A* (*PfgyrA*) gene, suggesting that the DNA binding specificity of PfARO is likely due to the AT-richness of the probe. This is a novel functional characteristic that has not been reported previously for any *P*. *falciparum* ARM containing protein and suggests a putative role for PfARO in gene regulation. This study describes for the first time a conserved *P*. *falciparum* ARM repeat protein with a high degree of functional versatility.

## Introduction

Apicomplexan parasites are the cause of enormous disease burden worldwide. The phylum Apicomplexa consists of numerous pathogenic protozoans such as *Plasmodium falciparum*, *Toxoplasma gondii*, *Theileria annulata* and *Neospora caninum*, which cause various diseases in both humans and a variety of economically important animal species. Among them, the human malaria parasite *P*. *falciparum* is an obligate intracellular protozoan with a complex lifecycle and a leading cause of malaria mortality reflected in around a million deaths annually [1]. Host cell invasion by the parasite is crucial for malaria pathogenesis. The virulence of *P*. *falciparum* is critically linked to the evolution of a large repertoire of invasion related proteins localized in specific apical organelles that ensure its entry into diverse host cells as well as its propensity to robustly multiply both at the liver and blood stages of the life cycle [2–4]. *P*. *falciparum* erythrocyte invasion is carried out by merozoites, which are small pear shaped invasive stages of the parasite containing specialized apical organelles (rhoptries, micronemes). The erythrocyte invasion process consists of a complex series of events culminating in an ordered discharge of the apical organelles and eventual entry of the parasite inside the host cell [2–4].

An analysis of the sequenced genomes of the Apicomplexan parasites of medical and veterinary importance has revealed the presence of a novel family of parasite proteins consisting of a unique Armadillo (ARM) repeat motif [5–9]. The physiological role of these ARM (Armadillo) repeat containing proteins remains to be completely elucidated with the functional characterization for only a few members reported till date [7, 8]. On the other hand, eukaryotic ARM repeat containing proteins have been demonstrated to be functionally versatile [9]. These proteins are characterized by the presence of a ~ 40 amino acid repeat that may exhibit minor variation in length [10]. These repeats comprising of 3 alpha-helices were identified for the first time in the Drosophila segment polarity protein, Armadillo [11]. Since then the repertoire of this family of proteins has been ever increasing and so is the portfolio of their diverse cellular functions. Tandem ARM repeat units in these proteins fold into a super-helix that gives rise to a protein-protein interaction platform, which has been primarily implicated in its multiple cellular roles [12]. Interestingly, these proteins are structurally conserved irrespective of their low sequence identity [13–15]. Key examples of armadillo domain containing proteins are the family of Beta-catenins that are known to have a dual function of both regulating cell–cell adhesion and gene transcription. The structure of these Beta-catenin like proteins is a key to their diverse functional role [10] that range from cell adhesion, protein degradation in the cytoplasm, nucleo-cytoplasmic transport, cell signaling to gene regulation in the nucleus [9, 12]. Mammalian Beta-catenin is itself central to the Wnt signaling pathway whereby in the presence of an appropriate stimulus, it enters the nucleus and in conjunction with transcription factors regulates the expression of genes responsible for cell proliferation and development [16–18]. ARM repeat containing proteins in plant systems have also received immense attention [12]. As noted earlier, these proteins have been extensively studied in multi-cellular eukaryotes, however it has remained largely unexplored in unicellular protozoans. Several putative ARM repeat containing proteins have been identified in the Apicomplexan genome databases [5]. A few are conserved across higher eukaryotes, while a number of them are specific to Apicomplexans itself. Importantly, the homologues of two conserved eukaryotic ARM repeat containing proteins, Importin-alpha and PF16, have been identified in *P*. *falciparum* [6] and *P*. *berghei* [7]. *P*. *berghei* PF16 stabilizes the flagellar central apparatus playing a crucial role in male gamete biology [7]. An eight Armadillo/β catenin-like repeat containing homologue of Importin-alpha also known as, Karyopherin alpha, was reported in *P*. *falciparum* [6].

Interestingly, one novel *P*. *falciparum* ARM repeat containing protein, PfARO (PF3D7_0414900/ PFD0720w), which is highly conserved across *Plasmodium spp* and Apicomplexan parasites, was reported to be targeted to the rhoptry membrane facilitated by lipid modifications at its N-terminus [19]. The *Toxoplasma* homologue of the protein has been shown to be crucial for the accurate positioning of the rhoptry at the apical end of the parasite, which in turn was demonstrated to be essential for host cell invasion by the *T*. *gondii* parasites [8]. In this study, we have discovered that the PfARO protein is not only targeted to the apical end of the parasite at the late asexual erythrocytic stages but also exhibits a nucleo-cytoplasmic shuttling similar to that observed for the ARM domain containing Beta-catenin proteins. Moreover, this stage specific nuclear enrichment was sensitive to the nuclear transport inhibitor, Leptomycin B (LMB).

We thus demonstrate for the first time a dual sub-cellular localization of *P*. *falciparum* ARO (PfARO), an Armadillo (ARM) repeat containing protein and the phenomenon of stage specific nucleo-cytoplasmic shuttling for any Apicomplexan parasite. Importantly, PfARO exhibits a specificity to bind AT rich DNA of *Pfgyr*A gene. This specific binding to AT rich sequence suggests a putative role of PfARO in DNA stabilization or gene regulation. Hence, taken together, our results report a *P*. *falciparum* beta-catenin like Armadillo repeat containing protein that exhibits a dynamic pattern of cellular localization and a functional DNA binding activity.

## Results

### Expression of the full-length recombinant PfARO protein and generation of specific PfARO antibodies

**Fig 1:**
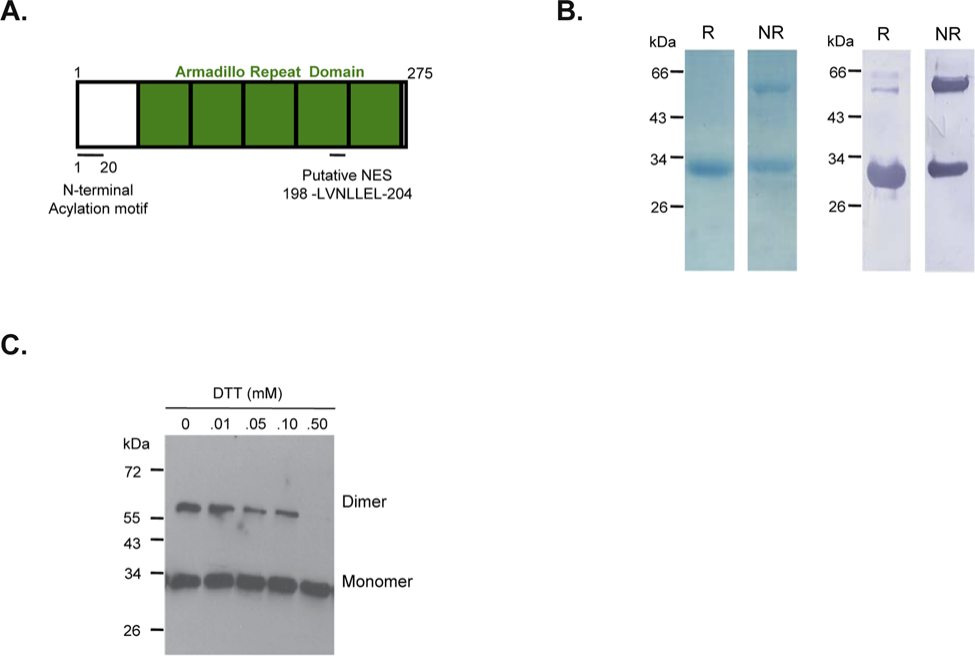
Expression and characterization of Recombinant PfARO (rPfARO). **A.** Schematic representation of the domain structure of *P*. *falciparum* ARO (PfARO) showing the N-terminal acylation region, ARM repeat domains and putative nuclear export signal. **B.** SDS-PAGE of purified recombinant rPfARO run under reducing (R) and non-reducing conditions (NR). PfARO under non-reducing condition shows a prominent band migrating at a dimeric position in addition to the monomeric form. Both monomeric and dimeric forms were recognized by anti-His antibodies in immunoblot analysis (right panel). **C.** Immunoblot analysis of recombinant PfARO using anti-His antibodies in the presence of increasing concentrations of the reducing agent, Dithiothreitol (DTT). The dimeric form of rPfARO diminished progressively with the increasing DTT concentrations suggesting a disulphide mediated homodimerization.

The domain architecture of PfARO (Fig 1A) revealed that the ARM repeats span entirely across the protein, with the acylation sites crucial for its rhoptry localization present at its N-terminus [19]. In addition, with the aid of the bioinformatics tool NetNES 1.1, we identified a putative leucine rich nuclear export signal located 70 amino acids from its C-terminus spanning the residues 198–204 (LVNLLEL) [20]. To enable characterization of PfARO, the full length protein was chosen for recombinant expression in *E*. *coli*. The full length PfARO open reading frame encoding 275 amino acids was PCR amplified from 3D7 cDNA and cloned into the T7 promoter based expression vector pET-24b. Recombinant PfARO (rPfARO) got expressed as a soluble protein in *E*. *coli* with a 6-His tag at its C-terminus. The 32 kDa recombinant PfARO protein was purified to homogeneity by metal affinity chromatography (Ni-NTA column) followed by size-exclusion chromatography. SDS-PAGE analysis of the purified rPfARO protein showed a monomeric protein with an expected mobility of ~32 kDa under reducing conditions while under non-reducing conditions an additional prominent ~60 kDa band was also observed suggesting that recombinant PfARO has a propensity to undergo cysteine mediated dimerization (Fig 1B). PfARO protein consists of an odd number (nine) of cysteine residues, suggesting that one cysteine may be involved in homo-dimerization.

Recombinant PfARO was identified both in its monomeric and dimeric forms in immunoblots using a specific anti-His tag antibody (Fig 1B), confirming expression of the full-length recombinant protein with the C-terminal His tag. Further, we observed that the dimeric form of rPfARO gradually disappeared in presence of increasing concentrations of the reducing agent, Dithiothreitol (DTT) (Fig 1C), further confirming that the dimerization occurs through cysteine mediated disulfide bonds. The two protein bands corresponding to the monomer and dimer were excised from the gel and subjected to trypsin digestion followed by LC-MS (liquid chromatography-mass spectrometry) analysis (Orbitrap VELOS PRO; Thermo Fisher Scientific). The LC-MS analysis of both the monomeric and dimeric forms revealed a large number of unique high-scoring peptides of PfARO thus confirming their identity as rPfARO (S1 Table).

**Fig 2:**
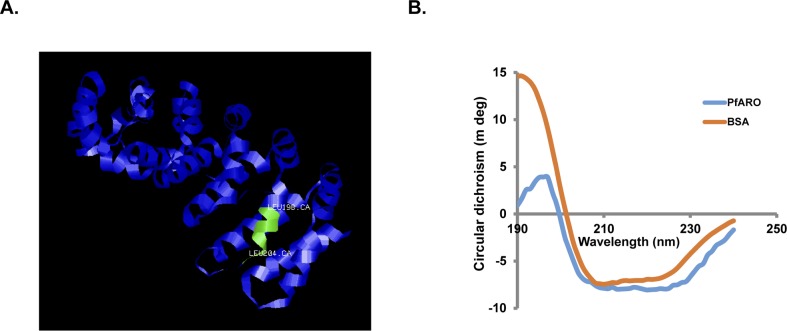
PfARO exhibits structural conservation with other ARM repeat containing proteins. **A.** Three-dimensional model of rPfARO predicted through homology modeling using Phyre2 software and visualized by Jsmol. The putative nuclear export signal (NES) is depicted in green. Model dimensions (Å): X:42.005 Y:58.467 Z:60.106. **B.** Circular dichroism analysis of recombinant rPfARO showed a helical composition consistent with the presence of tandem ARM repeats. The CD spectra of a known helical protein, BSA was run as a control.

ARM repeat containing proteins predominantly comprise of a helical secondary structure [13]. Three dimensional molecular modeling of PfARO also showed a predominantly helical secondary structure that is highly conserved among ARM repeat containing proteins (Fig 2A). The predicted NES of PfARO located ~70 amino acids from the C-terminus was observed to be on the surface of the protein and not buried inside (Fig 2A). Further, circular dichroism (CD) analysis of recombinant PfARO showed a helical composition (58%), which was consistent with the modeling data conforming to the presence of tandem ARM repeats (Fig 2B). As a control, CD analysis was performed with another known helical protein, Bovine Serum Albumin (BSA) for which 67% helicity was observed in accordance with that reported earlier [21] (Fig 2B). Mice and rabbits were immunized with recombinant PfARO to raise PfARO-specific antibodies. The specificity of the PfARO mice and rabbit antibodies was analyzed by immunoblotting studies to detect the recombinant rPfARO protein (S1A Fig). Both the mice and rabbit anti-PfARO antibodies detected the 32 kDa rPfARO at the expected size while the pre-immune antibodies failed to detect any protein (S1A Fig).

### Expression of native PfARO was detected in the parasite and culture supernatant

**Fig 3:**
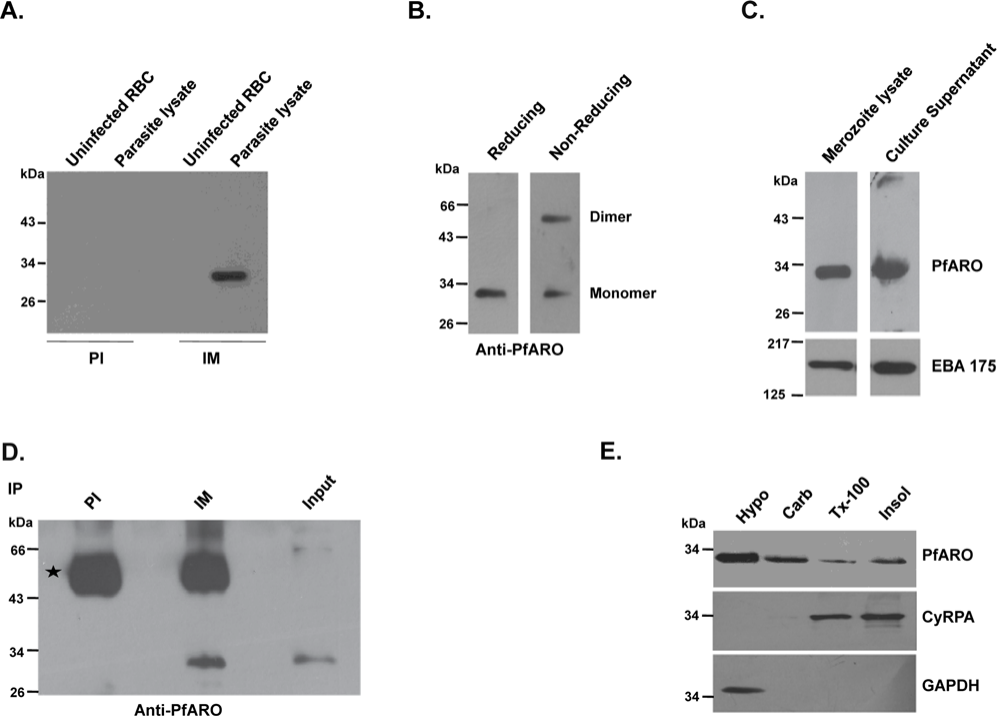
Expression of the native PfARO parasite protein and analysis of its solubility in the merozoite. **A.** PfARO antibodies specifically detected native PfARO at the expected molecular mass (~32 kDa) in detergent based lysates of schizont stage parasites. No cross-reactivity was observed with any other member of the ARO family of protein or with human erythrocyte proteins from the lysate of uninfected erythrocytes/red blood cells (RBCs). Immunoblot analysis under identical experimental conditions using pre-immune (PI) sera also did not exhibit any cross-reactivity by detecting any other parasite proteins. **B.** Under non-reducing conditions, native PfARO was also detected as a dimer(~60KDa) in addition to the monomeric protein (~32 KDa). **C.** Native PfARO was specifically detected by immunoblot analysis in a detergent based lysate of the merozoite stage parasites and culture supernatant. Native EBA-175, a key invasion protein ligand, was detected as a control in both merozoite lysate and culture supernatant. **D.** PfARO mouse antibodies (immune sera, IM) specifically immunoprecipitated native PfARO from detergent based lysates of late schizont stage parasites. Pre-immune sera failed to immunoprecipitate native PfARO. The immunoprecipitated native PfARO protein were detected in immunoblots using the same PfARO mouse sera that detected the mouse IgG heavy chain (denoted in the Fig by an asterisk). Input represents the lysate sample used for immunoprecipitation. **E.** PfARO was found to be present in the supernatants obtained from the *P*. *falciparum* parasites subjected to hypotonic lysis, sodium carbonate extraction, Triton X-100 extraction as well as the insoluble fraction. GPI anchored parasite protein, CyRPA, was found predominantly in the Triton X-100 detergent extracted and insoluble fractions suggesting that it was tightly membrane associated, while the cytosolic native PfGAPDH protein was as expected found to be in the supernatant following hypotonic lysis.

The anti-PfARO mice antibodies specifically detected native PfARO at the expected size of ~32 kDa in immunoblots using a detergent based schizont-stage parasite lysate while no cross-reactivity was observed either with uninfected RBC lysate or with the pre-immune control sera (Fig 3A). Immunoblot analysis of the parasite lysate under non-reducing conditions detected PfARO as a dimer consistent with the dimers observed with the recombinant protein preparation (Fig 3B). PfARO has been reported to be localized to the outer membrane of the apical organelle, rhoptry, which is known to harbour invasion related proteins that get secreted to the merozoite surface during the different steps of erythrocyte invasion. Further, the anti-PfARO antibodies detected the 32 kDa native PfARO in both detergent based lysates of free merozoites as well as culture supernatant (Fig 3C). As a control, antibodies against a key invasion ligand, EBA-175, also detected the presence of native PfEBA-175 in both the merozoite and culture supernatant. However, native PfARO did not exhibit erythrocyte binding activity (S2 Fig) and neither did its antibodies exhibit invasion inhibitory activity (S3 Fig).

Immunoprecipitation experiments followed by immunoblot analysis showed that the rPfARO antibodies were able to successfully immunoprecipitate the native PfARO protein from the schizont-stage parasite lysate (Fig 3D). The identity of the immunoprecipitated native PfARO protein was also confirmed by mass spectrometry that also validated the specificity of the PfARO antibodies. Pre-immune control antibodies did not immunoprecipitate native PfARO. Native PfARO was consistently detected with a significant high score between two independent experiments (S2 Table).

*P*. *falciparum* contains 10 ARM repeat containing proteins of which only PfARO has a molecular mass of 32 kDa with the other nine ranging from 38–288 kDa. The specific detection of native PfARO at the expected size of 32 kDa by immunoblotting of three different parasite preparations—schizont stage parasite lysate (Fig 3A and 3B) free merozoite lysate (Fig 3C), culture supernatant (Fig 3C) and immunoprecipitation (Fig 3D) followed by mass spectrometric confirmation (S2 Table) strongly suggests that our PfARO antibodies are highly specific and precisely recognize only the native PfARO parasite protein.

A solubility assay was performed using free merozoites to ascertain the association of PfARO with the rhoptry membrane as reported earlier [19]. The free merozoites were subjected to lysis in a sequential manner as reported earlier [19]. First the parasites were subjected to hypotonic lysis with water including mechanical shear, followed by Na_2_CO_3_ and Triton X-100 extraction. Consistent with a previous report [19], PfARO was detected in all three fractions (soluble, carbonate and Triton X-100) but predominantly observed to be present in the supernatants derived from hypotonic lysis and Na_2_CO_3_ extraction with only a small amount of protein detected in the supernatant from Triton X-100 extraction. Our data suggests that PfARO is peripherally attached with the rhoptry membrane with a low affinity that can be broken by hypotonic lysis and Na_2_CO_3_ extraction (Fig 3E). As controls, the cytosolic GAPDH protein [22] was detected only in the supernatant from hypotonic lysis, while the membrane associated GPI-anchored protein (CyRPA) was detected only in the Triton X-100 detergent derived and insoluble fractions as reported earlier [23].

### PfARO exhibits dynamic localization and nucleo-apical shuttling during the intra-erythrocytic life cycle

**Fig 4:**
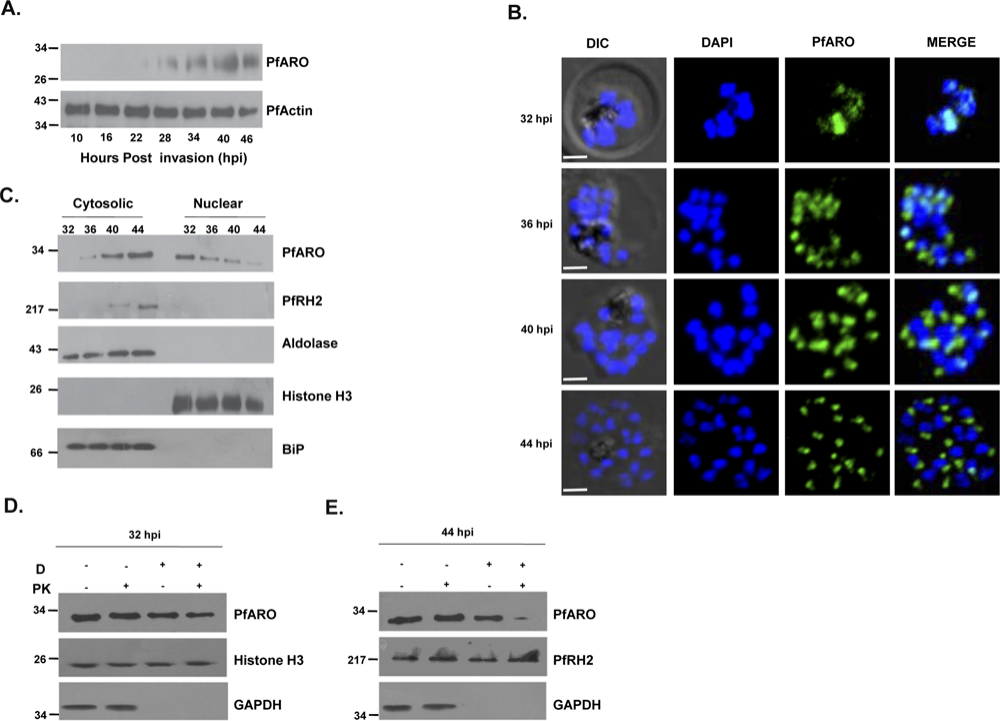
Native PfARO exhibits nucleo-apical shuttling. **A.** Stage specific expression analysis of native PfARO during the intra-erythrocytic asexual 48 hour cycle by immunoblotting using specific PfARO antibodies. PfARO expression is initiated at early schizont stages (30–32 hours post infection) and peaks at late schizont stages (44–48 hpi). Expression of the constitutive parasite protein, PfActin was analyzed as a control. **B.** Confocal immunofluorescence imaging using PfARO specific antibodies detected predominant expression of native PfARO in the nucleus during the early schizont stage parasites, which decreased with the intra-erythrocytic growth of the parasite. At later stages, native PfARO was observed to be predominantly outside the nucleus (Scale bar, 2 μm). **C.** Stage specific nuclear fractionation followed by immunoblot analysis was consistent with the imaging results as It native PfARO was detected in the nuclear fraction in early schizont stage (32 hpi) while it is mostly non-nuclear or cytosolic at the late schizont stages (44 hpi). As controls, Histone H3 was found exclusively in the nuclear fraction; Aldolase was found in the cytosolic fraction as was the apically localized rhoptry protein, PfRH2. The ER resident protein marker Bip was also found to be in the cytosolic fraction. **D**. Native PfARO remained intact and protected from Proteinase K (PK) degradation in digitonin (D) permeabilized early schizont stage parasites (32 hpi) but was susceptible to PK action at the late stages (44 hpi). Other organellar proteins like the nuclear protein Histone H3 and rhoptry protein PfRH2 also remained protected from PK action in the permeabilized parasite infected erythrocytes, while the cytosolic protein GAPDH was lost on permeabilization.

PfARO has earlier been reported to be localized to the rhoptry in late schizont stage parasites [19]. We investigated its expression and localization during the different stages of the 48 hour intra-erythrocytic life cycle by immunofluorescence staining and immunoblotting. Expression of native PfARO initiates at the early schizont stage with peak expression observed at the late schizont stage (Fig 4A). PfARO exhibited a predominant nuclear localization during the early schizont stages, while as the parasite underwent intra-erythrocytic development it exhibits cytosolic (non-nuclear) localization at the apical pole of the parasites at the late schizont stage (Fig 4B).The staining of the PfARO antibodies were specific as the pre-immune antibodies failed to detect any background or cross-reactive signal (S1B Fig). This unique pattern of dynamic localization during the different stages of the asexual blood-stage life cycle suggests that PfARO may be undergoing a nucleo-apical shuttling. The differential localization was also confirmed by sub-cellular fractionation experiments in which infected erythrocytes from different time points of the asexual blood-stage life cycle ranging from early to late schizogony were subjected to nucleo-cytosolic fractionation. Immunoblot analysis showed that PfARO was predominantly in the nuclear fraction during the early schizont stages, which progressively decreased with the parasite’s growth and was accompanied with a concomitant increase in the cytosolic expression of PfARO during the late schizont stages (Fig 4C). Here, ‘cytosolic’ pertains broadly to a non-nuclear localization. The purity of the nuclear and cytosolic fractions were validated by analyzing the presence of standard marker proteins known to be located in the nucleus or cytosol of *P*. *falciparum* parasites. Histone H3 was analyzed as a marker for the nuclear fraction and was exclusively detected only in the nuclear extracts (Fig 4C). Whereas, three known *P*. *falciparum* non-nuclear proteins, PfRH2 (rhoptry), PfAldolase (cytosol) and PfBip (ER) were exclusively found in the cytosolic fraction (Fig 4C) supporting a clear distinction between nucleus and the rest of the cell in the fractionation experiment (Fig 4C).

In a Proteinase K protection assay, we also demonstrated that PfARO exhibits a differential sensitivity to Proteinase K treatment during the different stages of the blood-stage life cycle that correlates with its dynamic localization (Fig 4D). The permeabilization of the parasite plasma membrane by digitonin and subsequent Proteinase K treatment leads to the degradation of all exposed parasite proteins. Only the parasite proteins protected by any organelle membrane would remain intact. Interestingly, PfARO remained intact after Proteinase K treatment at the early schizont stages whereas it was observed to be degraded during late schizonts (Fig 4D). The resistance of PfARO during the early schizont stage is consistent with its localization within the nucleus that confers protection from proteinsase K treatment. Similarly, the H3 histone protein in the nucleus also remained protected, while the cytosolic GAPDH protein got released on digitonin treatment implying the specific permeabilization of the parasite plasma membrane (Fig 4D). During the late schizont stage, PfARO is localized on the outer rhoptry membrane (cytoplasmic face) exposing it to the enzymatic treatment leading to its degradation (Fig 4E) as reported earlier [19]. On the other hand, the internal rhoptry protein PfRH2 [24] that was not exposed to Proteinsase K treatment remained protected (Fig 4E). Our data from this assay further substantiates our observation of the dual localization of PfARO during the different stages of the asexual erythrocytic life cycle.

**Fig 5:**
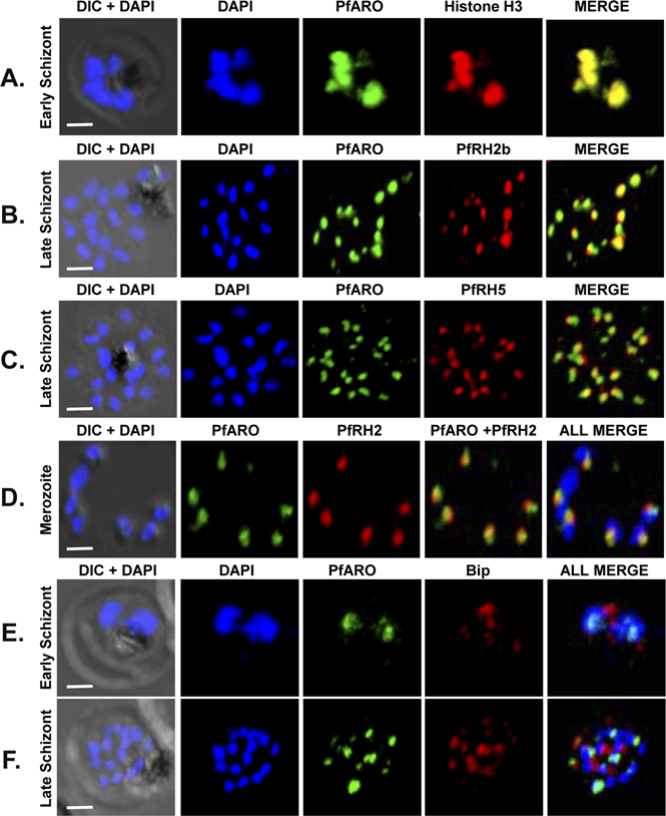
Colocalization of native PfARO with nuclear marker at early schizont stage and rhoptry markers at late schizont stage as demonstrated by confocal immunofluorescence imaging. **A.** PfARO co-localizes with the nuclear marker protein, Histone H3 at the early schizont stages. **B.** PfARO is apically located at the late schizont stages as demonstrated by its co-localization with rhoptry neck marker PfRH2. **C.** PfARO is apically located at the late schizont stages as demonstrated by its co-localization with rhoptry bulb marker PfRH5. **D.** PfARO co-localizes with PfRH2 at the apical end of the parasites in free merozoites. **E.** PfARO does not co-localize with the ER resident protein Bip either at early or late schizont stages of the parasite. DAPI was used for staining the nucleus. (Scale bar, 2 μm).

PfARO co-localized with the nuclear marker protein, histone H3, confirming its nuclear localization at the early schizont stage (Fig 5A). Consistent with previous reports [8,19], PfARO co-localized with the rhoptry marker proteins, PfRH2/PfRH5 [24, 25] during the late-schizont stages (Fig 5B and 5C) as well as free merozoites (Fig 5D), confirming its known localization on the outer rhoptry membrane. We also observed that an ER resident protein marker PfBip did not co-localize with PfARO at early schizont stages when it is localized in the nucleus ruling out the possibility that this nuclear localization may simply be the protein’s initial trafficking through the ER, which is in close proximity to the nucleus (Fig 5E). Even in the late stage schizonts when the rhoptries have matured, PfARO exhibits a distinct localization from that of PfBip further suggesting that there is no cross-reactvity between their signals (Fig 5F).

**Fig 6:**
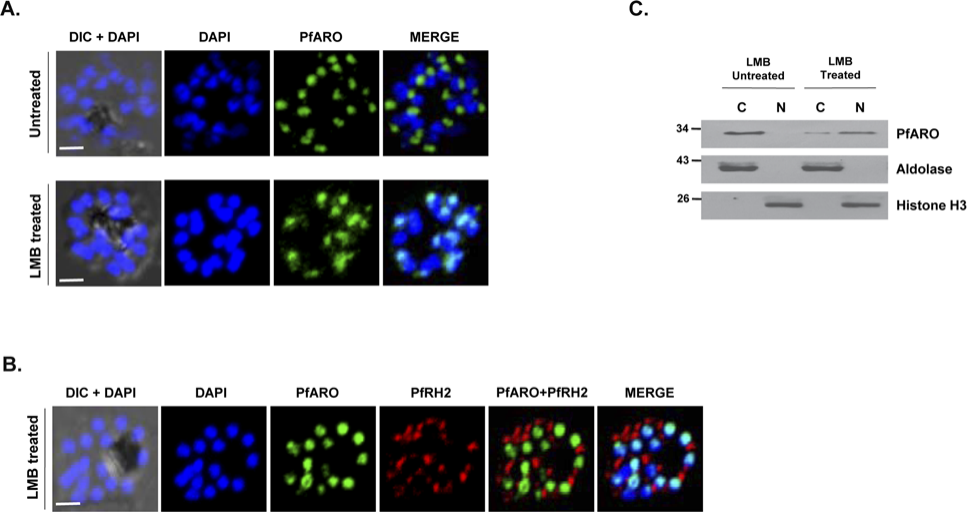
Leptomycin B (LMB) abrogates PfARO export out of the nucleus. **A.** Localization of PfARO in the parasite at late stages is affected in presence of the nucleo-cytoplasmic transport inhibitor LMB. In untreated parasites PfARO is apically located at late schizont stages. Upon treatment with LMB, PfARO remains within the nucleus as shown by confocal immunofluorescence analysis. DAPI was used for staining the nucleus and it is clear that the PfARO signal colocalizes with the DAPI staining. **B.** Under the same experimental conditions, the localization of the rhoptry protein PfRH2 remains unaffected. (Scale bar, 2 μm). **C**. Sub-cellular fractionation followed by immunoblot analysis further confirmed the enrichment of PfARO in the nuclear fraction in presence of LMB with respect to untreated parasites. Histone H3 and aldolase are used as nuclear and cytosolic markers, respectively.

A putative leucine rich nuclear export signal (NES) was identified in PfARO spanning the residues 198–204 (LVNLLEL) through a bioinformatics analysis using NetNES 1.1 [20]. Transport of NES bearing proteins is primarily mediated through the CRM1 (chromosomal maintenance 1) or exportin protein [26]. A putative exportin-1 homolog has been reported in the *Plasmodium* genome. Interestingly, a fungal metabolite, Leptomycin B (LMB) has been established as an active inhibitor of exportin 1 mediated nuclear transport of NES bearing proteins [27, 28]. LMB treatment of trophozoite stage parasites caused nuclear accumulation of PfARO even at late schizont stages where it is normally localized to the apical pole of the parasites (Fig 6A) as demonstrated by immunofluorescence analysis. Under similar experimental conditions, the localization of the rhoptry protein PfRH2 was unaffected and remained apically localized at the late schizont stage (Fig 6B) suggesting that the formation of the rhoptry organelle was not affected by LMB treatment. Sub-cellular fractionation also confirmed our observation as PfARO expression levels were enriched in the nuclear fraction of LMB treated schizont-stage parasites compared to untreated parasites where it was predominantly in the cytosolic fraction (Fig 6C). Taken together our results suggest that the export of PfARO from the nucleus to cytoplasm is an NES and Exportin-dependent process.

### PfARO exhibits DNA binding activity

**Fig 7:**
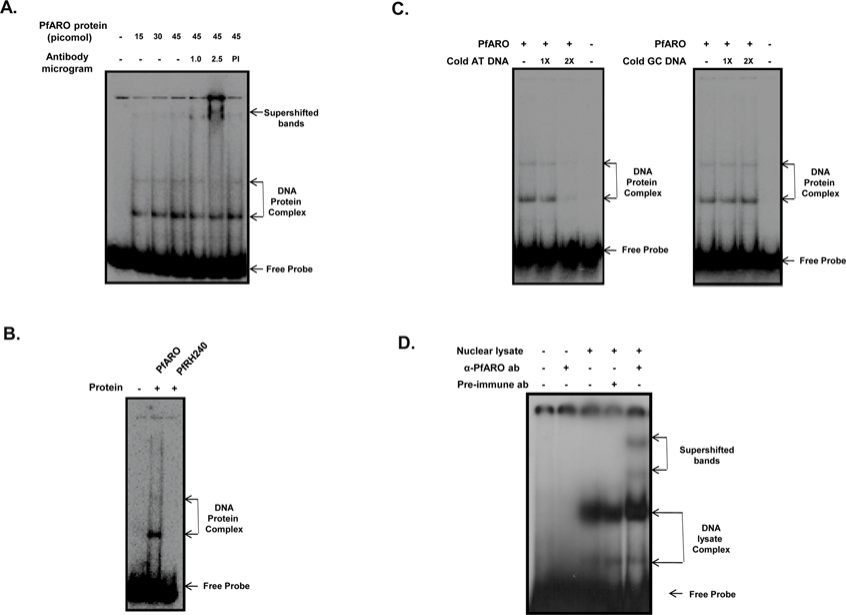
PfARO exhibits DNA binding activity. **A.** Purified full length recombinant PfARO binds to double stranded radiolabeled DNA as demonstrated by the Electrophoretic Mobility Shift assay (EMSA). The retardation of the DNA-protein complex with respect to the free probe is clearly shown. A super-shift in the mobility of the DNA-protein complex was specifically observed in the presence of the PfARO antibodies while no mobility shift was detected in presence of pre-immune sera. **B.** Recombinant PfRH2_40_ protein does not bind to double stranded radiolabeled DNA probe under the same experimental conditions in which rPfARO exhibits DNA binding activity. **C.** A competition assay was performed using cold AT-rich or GC-rich competitor DNA probes at varying concentrations (1X, 2X). The cold AT rich DNA abrogated the DNA binding of PfARO while the presence of the GC rich cold DNA had no effect on the DNA binding activity of PfARO. **D.** Native PfARO present in the nuclear lysate specifically bound the double stranded radiolabelled DNA as demonstrated by the super-shifted complex only in the presence of PfARO specific antibodies. The PfARO specific antibodies did not exhibit any affinity for the radiolabelled probe. No super-shift was observed with the control pre-immune sera.

Beta-catenin-like proteins are known to be important signaling molecules that mediate transcriptional activation [17]. To elucidate whether PfARO that displays beta-catenin like characteristics, is capable of directly binding to DNA, we performed Electrophoretic Mobility Shift Assays (EMSAs) using full length recombinant rPfARO and a double-stranded AT rich *P*. *falciparum* DNA probe [29]. Recombinant rPfARO specifically bound with the double stranded DNA probe as another recombinant protein rPfRH2_40_, representing the erythrocyte binding domain of the PfRH2 parasite adhesion protein [24], failed to exhibit any DNA binding activity (Fig 7A and 7B). Increasing concentrations of rPfARO formed a DNA–protein complex that migrated at a slower rate and could also form higher order complexes (Fig 7A). The DNA protein complex further displayed a specific super-shift in the presence of the anti-PfARO antibody while no such super-shift was observed with the pre-immune antibodies (Fig 7A).

The *Plasmodium* genome is uniquely 80% AT rich and to further confirm the binding specificity of PfARO for AT-rich DNA, we performed a competitive EMSA in the presence of increasing concentrations of cold AT-rich or GC-rich DNA [29]. Cold GC-rich DNA had no effect on the DNA binding specificity of PfARO, whereas cold AT-rich DNA competitively inhibited the DNA binding of PfARO, thus confirming the binding specificity of PfARO towards AT-rich DNA of *PfgyrA* (Fig 7C). Similar EMSA experiments were performed with *P*. *falciparum* nuclear extracts to demonstrate the binding specificity of the native PfARO parasite protein towards AT rich *Plasmodium* DNA. DNA-protein complex formation was observed only in the presence of the nuclear extract (Fig 7D) with no complex formation observed between the PfARO antibodies and radiolabeled DNA probe (Fig 7D). The DNA-protein complexes represent the binding of the radiolabeled DNA with a number of *P*. *falciparum* protein in the nuclear extract. The specificity of native PfARO was confirmed by the specific super-shift detected only in the presence of anti-PfARO antibodies and not with the pre-immune antibodies, suggesting that the native PfARO protein exhibits specific DNA binding activity (Fig 7D). Our data has demonstrated for the first time that PfARO, an Apicomplexan parasite Armadillo domain containing protein could exhibit direct DNA binding activity.

## Discussion

Our data provides the first evidence of stage specific nucleo-apical shuttling of an ARM domain containing *P*. *falciparum* protein, PfARO, which had previously been shown to be apically localized in protozoan parasites. Apicomplexan ARO proteins have been demonstrated to be localized to the rhoptries and this organelle targeting is mediated through lipid modifications at its N-terminus such as palmitoylation and myristoylation [8, 19, 30]. The similarity of PfARO with beta-catenin like molecules goes beyond just sharing the ARM repeat motif as it is also localized in both the nucleus and cytoplasm [17]. While, PfARO shares a low overall identity with other eukaryotic ARM domain containing proteins, it does exhibit a significant structural conservation with them. PfARO lacks a classical nuclear localization signal (NLS), which is again characteristic of beta-catenin like molecules, whose nuclear import is NLS independent [31, 32]. In fact, the major nuclear import factor, Karyopherin-alpha, has been shown to carry these ARM repeat proteins facilitating their trafficking to the nucleus [33].

PfARO is not constitutively localized in the nucleus, but instead its presence in the nucleus is stage specific. Our high resolution microscopy and sub-cellular fractionation studies confirmed its presence in the nucleus at the early schizont stages when parasite genomic DNA replication is reported to occur at a high efficiency [34]. Targeting of PfARO to the rhoptry at the late schizont stages is consistent with previous studies in *P*. *falciparum* and *Toxoplasma gondii*, where it has been demonstrated to play a crucial role in apical rhoptry positioning and consequently in host cell invasion [8, 19].

We further demonstrated that the nucleo-cytosolic export of PfARO is mediated through the CRM1 mediated pathway as PfARO shuttling to the cytosol was sensitive to Leptomycin B (LMB), a known inhibitor of the CRM1 mediated export pathway [28]. CRM1 mediated translocation requires a leucine rich nuclear export signal (NES) in its cargo and bioinformatics analysis has identified a putative canonical leucine rich NES in PfARO. Our data is consistent with previous studies in which another ARM domain containing protein, APC, was demonstrated to exhibit nucleo-cytoplasmic shuttling that was mediated through the CRM1 mediated nuclear export pathway [35–37].

We have further demonstrated that within the nucleus, PfARO exhibits direct DNA binding activity. Unlabeled cold AT rich DNA competitively inhibited PfARO binding to an AT rich *PfgyrA* DNA probe, which was unaffected by an unlabeled GC rich probe. The binding of PfARO to the Gyrase A probe is likely due to the AT-richness of the probe rather than its specific sequence. The preferential binding of PfARO to AT rich DNA is a property that has also been previously demonstrated for another mammalian ARM repeat containing protein, Adenomatous Poliposis Coli (APC) [38].

The ability of PfARO to be present in different sub-cellular locations during different time points of the intra-erythrocytic life cycle of the parasite displays its biological versatility that is characteristic of the beta-catenin proteins and family of ARM repeat containing proteins. The PfARO armadillo repeat containing protein is conserved across different *Plasmodium* species indicating that its multifunctional nature may be crucial for the parasite [9].

Our data brings to the forefront a *P*. *falciparum* ARO protein (PfARO) as an example of an Apicomplexan parasite ARM repeat containing protein that belongs to a class of divergent beta-catenins, which in spite of sharing a low sequence identity with canonical beta-catenins exhibits a structural conservation and appears to fulfill their key beta-catenin like function. This is similar to the case of the *C*. *elegans* SYS-1 protein, which also displays low sequence identity with canonical beta-catenins but functionally behaves as a beta-catenin [15]. Similarly, an *Arabidopsis* armadillo repeat containing protein, ABAP1, was previously demonstrated to be part of a unique signaling network, which controls cell cycle progression through its participation in transcriptional control of DNA replication genes and its direct association with DNA pre-replication complex proteins [39]. ARM repeat proteins have not been shown to have a function in gene expression or signaling in unicellular organisms but do have a conserved signaling function in *Dictyostelium*, a unicellular free-living amoeba that undergoes multi cellular developmental program. In fact, the *Dictyostelium* beta-catenin homologue ‘Aar’ has a dual structural and signaling role in intercellular adherence and junction formation [40]. Aar mediates the spore-cell differentiation and pre-spore gene expression [40].

PfARO is the first Apicomplexan parasite specific protein that has been demonstrated to exhibit features and characteristics similar to that of beta-catenin like molecules, which are implicated in signaling pathways that participate in a variety of biological processes including altering gene expression to altering cytoskeletal polarity [18, 32]. While, the role of PfARO in rhoptry positioning and consequent erythrocyte invasion has been established, it will now be interesting to further investigate its functional properties such as its putative role in gene regulation or DNA metabolism.

A potential role of PfARO as a transcriptional activator could be speculated as it is consistent with its specific binding activity to the AT rich DNA of *PfgyrA*. Regulatory motifs are predicted to be more AT-rich than other eukaryotic *cis*-acting motifs, given that the AT-content in the intergenic region of *P*. *falciparum* approaches 90% [41]. Beta-catenin like proteins in conjunction with other transcription factors are known to activate target genes involved in cell growth and proliferation in various organisms [17,18]. The Beta-catenin or Armadillo protein mode of transcriptional activation is mostly mediated through the upstream A-T rich promoter region of the regulated target genes, which acts as a binding site for the transcription factor [16]. Since Beta-catenin proteins also share ARM repeats, it is probable that PfARO may also similarly play a putative role in transcription regulation considering that it is localized in the nucleus during early schizont stages. Our data has provided interesting insights on PfARO and forms the basis for further in depth investigations to elucidate its precise physiological function and significance of its nucleo-apical shuttling.

## Materials and Methods

### Parasite Culture

*P*. *falciparum* (strain 3D7) was cultured in human RBCs as described previously [23]. The parasites were synchronized by sorbitol treatments [42].

### Cloning, Expression and Purification of recombinant PfARO

PfARO (plasmoDB ID PF3D7_0414900) was PCR amplified with cDNA template which had been prepared from 3D7 total RNA using the single-strand cDNA synthesis kit (Life Technologies), using primers TGTATGGAATTCCATATGGGAAATAATTGCTGTG (forward) and CCGCTCGAGATCCGTTAGTCTCAATAAG (reverse) carrying *Nde*I and *Xho*I sites respectively. The amplified fragment was cloned into pET24b vector. Soluble full length recombinant C-terminal 6X his tagged protein was obtained after BL21 (DE3) Codon+ *E*. *coli* cells transformed with the construct were induced with 0.3 mM IPTG at 16°C for 12–16 hours. The recombinant protein was purified by metal affinity chromatography using Ni-NTA resin (Qiagen, USA). The purified protein was dialyzed against 1X PBS and further purified to homogeneity using size exclusion chromatography (GE, Superdex-200 column). The identity of the recombinant protein was confirmed by mass spectrometry (LC-MS), as well as by immunoblotting using anti-His antibodies.

### Antibody production

The animal experiments described below were approved by the ICGEB Institutional Animal Ethics Committee (IAEC Reference No. MAL-89). ICGEB is licensed to conduct animal studies for research purposes under the registration number 18/1999/CPCSEA (dated 10/1/99).Animal immunizations were done as reported previously [43, 44]. Briefly, mice and rabbit were immunized intramuscularly with 25 μg and 300 μg of the recombinant protein respectively. The proteins were emulsified with complete Freund’s adjuvant (Sigma, St. Louis, MO) on day 0 immunization followed by two boosts emulsified with incomplete Freund’s adjuvant on day 28 and 56. The sera were collected on day 70.

### Electrophoretic Mobility Shift Assay (EMSA)

EMSA showing DNA binding activity of PfARO was performed as described earlier [29, 36]. DNA binding reactions were carried out in a 25μl reaction volume containing DNA binding buffer (10 mM Tris-Cl [pH 7.5], 100 mM KCl, 5 mM MgCl_2_, 2 mM dithiothreitol, 6% glycerol, 50 μg/ml bovine serum albumin) and different concentrations of protein incubated with appropriately radiolabeled [γ^-32^P]-ATP double stranded DNA fragment from the *P*. *falciparum* Gyrase A gene [29, 36]. The radiolabelled probe and AT-rich DNA (150 bp) was obtained by PCR amplification of a DNA fragment containing PfGyrA sequence using the primers (5’-CGGGATCCTTCAAGAGTAATCGTTCAAC-3’, 5’-TTTTATATCATTTAATTCATATGT-3’) [29]. GC-rich DNA (242 bp) was obtained by PCR amplification of a part of the tetracyclin resistance (tetr) gene from plasmid pBR322 using the primers (5-’CAAGCCGTCGACACTGGTCCCGCCA-3’, 5’-CGCGAGGGGAAT CCTTG AAGCTG-3’) [29]. The DNA binding gels were analyzed using Phosphoimager Storm 865 (GE) using the aid of the software ImageQuantTL (GE).

### Immunofluorescence staining of parasites and confocal microscopy

Parasite smears were fixed in pre-chilled methanol (-20°C) and were air dried and blocked using 3% BSA in PBS for 2 hours at room temperature (~25°C). Slides were incubated at room temperature (~25°C) for 1 hour with different antibodies at the following dilutions: anti-PfARO mouse sera (1:100), anti-PfRH2b [24] (1:50) and anti-PfRH5 [25] rabbit sera (1:100), anti-Histone H3 rabbit sera (1:50) (Abcam) and anti-Bip antibodies (1:100). Slides were then washed and incubated with secondary antibodies conjugated with Alexa Fluor 488/Alexa Fluor 594. The slides were washed and mounted in ProLong Gold antifade reagent with 4',6'-diamidino-2-phenylindole (DAPI) (Invitrogen) and were viewed on a confocal microscope (N-SIM, Nikon, Japan). The images were processed using the Nikon NIS Elements AR4.13.04 software.

### Nuclear and cytosolic fractionation and extract preparation

Nuclear and cytosolic extracts were prepared as previously described [45, 46] with some modifications. Parasites were isolated from infected erythrocytes by saponin lysis, re-suspended in 1 ml of lysis buffer (10mM HEPES pH 7.9, 10mM KCl, 0.1mM EDTA, 0.1mM EGTA, 1mM DTT, 0.65% NP-40) supplemented with protease inhibitors (Complete, Roche) and incubated for 30 min at 4°C. The lysate was centrifuged for 10 min at 14,000 rpm at 4°C. The supernatant representing the cytosolic fraction was recovered, aliquoted and stored at -80°C. The nuclei pellet was washed three times with phosphate-buffered saline (PBS) and then re-suspended in 100 μl of extraction buffer (20mM HEPES pH 7.9, 400mM NaCl, 1mM EDTA, 1mM EGTA, 1mM DTT) supplemented with protease inhibitors and incubated with vigorous shaking for 30 min at 4°C. The preparation was then centrifuged for 10 min at 14000 rpm at 4°C. The supernatant representing the nuclear fraction was recovered, aliquoted and stored at -80°C. The purity of the extracts was analyzed by immunoblotting, in which the extracts were probed with antibodies (Abcam) against the marker proteins for different cell compartments, anti- histone H3 (nucleus) and anti-aldolase (cytosol).

### Treatment of parasite with nucleo-cytoplasmic transport inhibitor LMB

The treatment of parasite with LMB was carried out using a modification of a protocol described earlier [47]. Briefly, synchronized late trophozoite stage parasites were treated with 25ng/ml of LMB (Sigma) for 4 hours. Parasites were retrieved at late schizont stages for immunoblot analysis or smears were prepared for immunofluorescence analysis.

### Homology modelling

Three-dimensional model of PfARO was created by protein homology modelling using Phyre2 (www.sbg.bio.ic.ac.uk/phyre2/) software and was visualized on JSmolPdbViewer. 6 templates (PDB c3nmwA, c2w3cA, c3tt9A, d1xqra1, c4db9A, c1xqrA) were selected to model the protein based on heuristics to maximize confidence, percentage identity and alignment coverage. 18 residues were modeled by ab initio [48].

### Proteinase K protection assay

This assay was carried out as per the methodology described earlier [19]. Saponin extracted parasites were resuspended in a buffer containing 0.6 M sorbitol, 20 mM Tris–HCl pH 7.5 and 2 mM EDTA. This suspension was aliquoted in different tubes and treated in presence or absence of 0.02% of digitonin (Sigma) for 10 minutes on ice, followed by centrifugation and removal of supernatant. Aliquots were further subjected to treatment in presence and absence of Proteinase K (0.1mg/ml) in the same buffer (0.6 M sorbitol, 20 mM Tris–HCl pH 7.5 and 2 mM EDTA) for 30 minutes on ice. Proteinase K was terminated by adding cold trichloroacetic acid. All the samples were centrifuged at 14000 rpm for 10 minutes, followed by an acetone wash. The samples were dried and reconstituted in TE and finally prepared for immunoblot analysis.

### Isolation of merozoites and solubility assays

The free merozoites were isolated as described earlier [23]. Briefly, the synchronized mature schizonts were allowed to rupture. The free merozoites were separated from erythrocytes by centrifugation at 2000 X g for 5 min. The pellet was discarded and the supernatant was again centrifuged at 4000 X g for 10 min to pellet the merozoites. The solubility assay protocol was adapted from solubility assays described previously [19]. Briefly, the sequential extraction was done; starting with hypotonic lysis in double distilled water, followed by freeze-thaw and mechanical disruption. The supernatant was saved and the pellet was resuspended in freshly prepared 0.1 M Na2CO3 and kept on ice for 30 min to extract peripheral membrane proteins. After centrifugation at 14000 rpm for 5 minutes, the supernatant was removed and pellet was washed with PBS. The washed pellet was further subjected to extraction for 30 min with 1% Triton X-100 and centrifuged at 14000 rpm for 5 min to obtain the integral membrane protein fraction in the supernatant. The final pellet was washed with PBS and resuspended in PBS containing the insoluble fraction. All the extracts were subjected to immunoblot analysis using specific antibodies.

## Supporting Information

S1 Fig Specificity of antibodies raised against recombinant rPfARO. (TIF)

S2 Fig PfARO does not exhibit erythrocyte binding activity. (TIF)

S3 Fig PfARO antibodies do not exhibit invasion inhibitory activity. (TIF)

S1 Table List of Unique Peptides generated from the Mass Spectrometric analysis (LC-MS) of the trypsin digested recombinant PfARO protein (monomeric band and dimeric band). (XLS)

S2 Table Mass spectrometric identification of native PfARO from immunoprecipitation elutes using specific antibodies. (XLS)

S1 Text Description of supplementary methods and figure legends (S1 Fig, S2 Fig, S3 Fig). (DOC)
