# Supplementary figures and images for: Evidence for the Nucleo-Apical Shuttling of a Beta-Catenin Like Plasmodium falciparum Armadillo Repeat Containing Protein
Source: PLoS One. 2016 Feb 1;11(2):e0148446. doi: 10.1371/journal.pone.0148446 (PMC4734682; doi:10.1371/journal.pone.0148446)

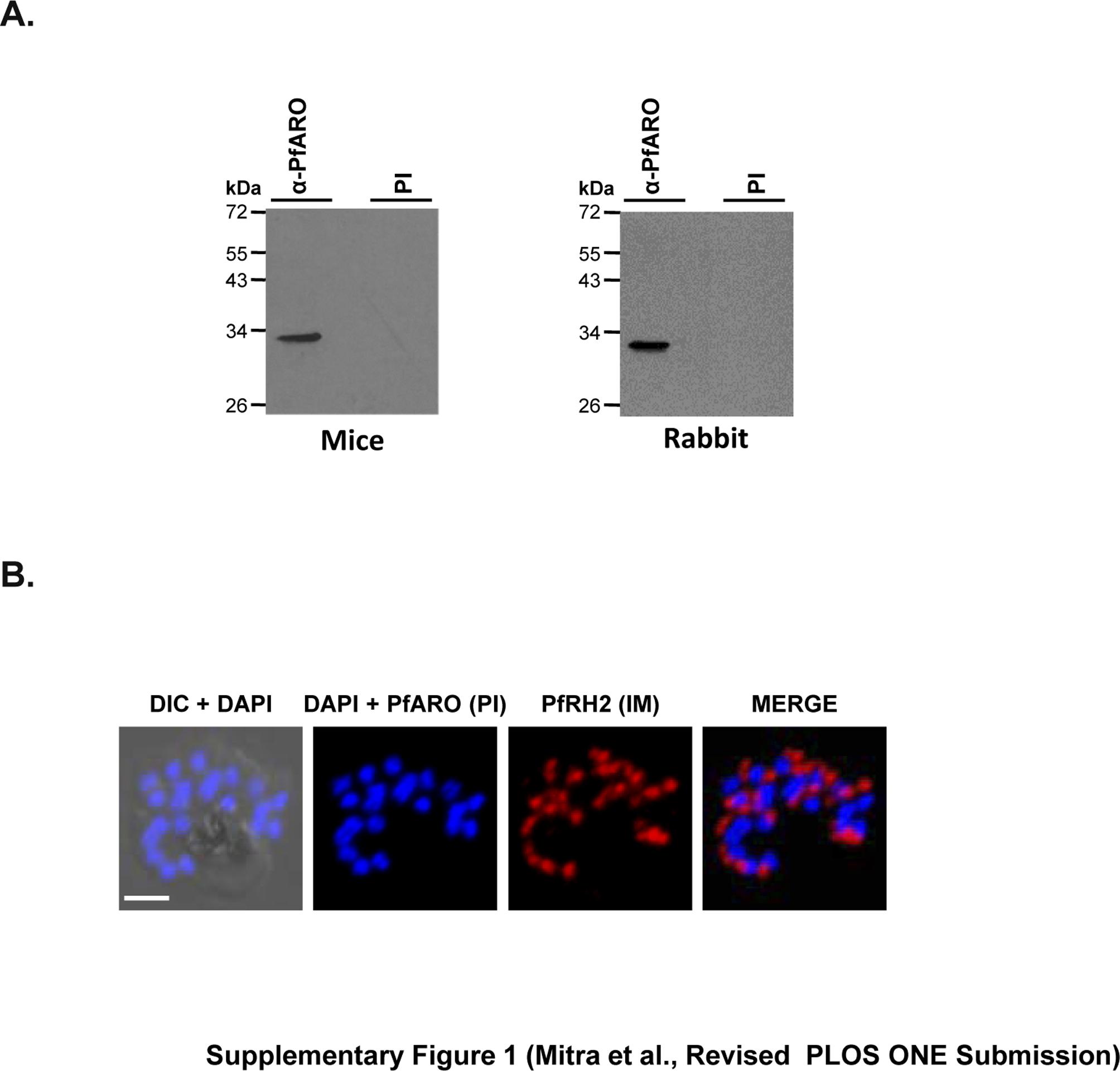

Supplement: S1 Fig — (TIF) [file pone.0148446.s001.tif]

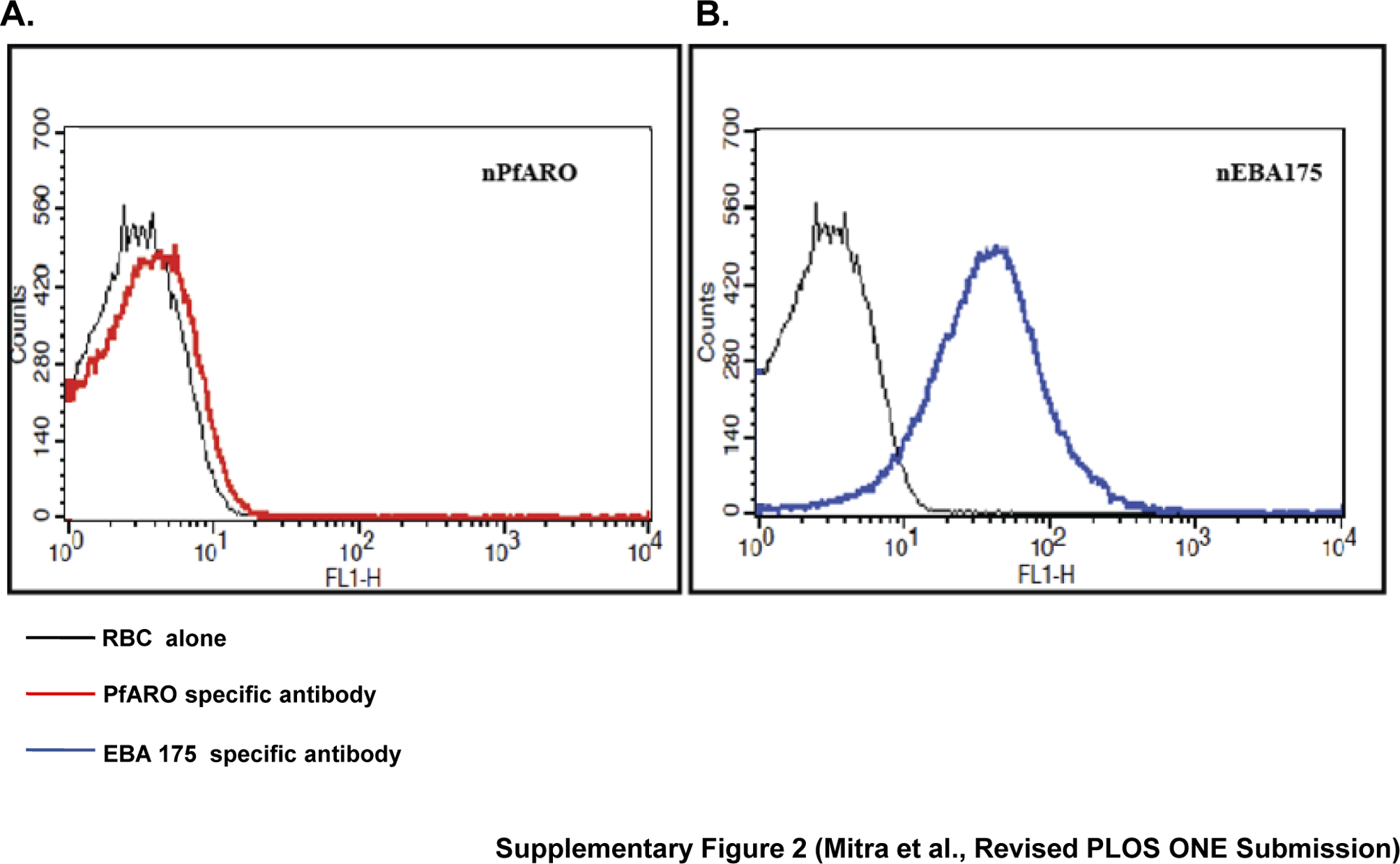

Supplement: S2 Fig — (TIF) [file pone.0148446.s002.tif]

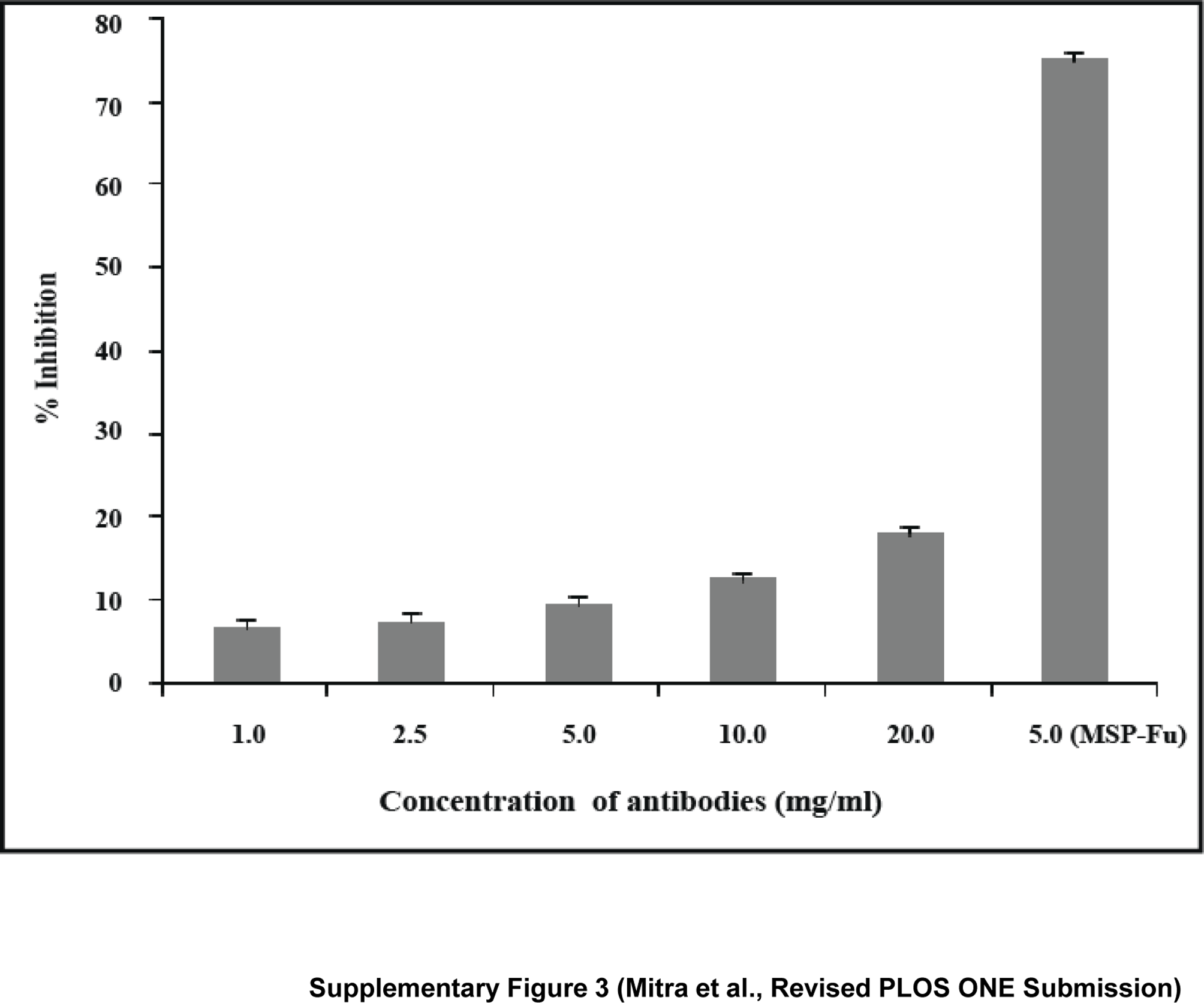

Supplement: S3 Fig — (TIF) [file pone.0148446.s003.tif]
