# Supplementary material for: Evidence for the Nucleo-Apical Shuttling of a Beta-Catenin Like Plasmodium falciparum Armadillo Repeat Containing Protein
Source: PLoS One. 2016 Feb 1;11(2):e0148446. doi: 10.1371/journal.pone.0148446 (PMC4734682; doi:10.1371/journal.pone.0148446)
Supplement: S1 Text — (DOC) [file pone.0148446.s006.doc]

**Supplementary Text**

**Methods**

**Mass Spectrometric analysis of purified recombinant protein**

In-gel digestion of the purified recombinant protein band was done to ascertain its identity. The corresponding protein bands from the coomassie stained SDS-PAGE gel were excised, reduced, and alkylated. Proteins were digested overnight with trypsin (Promega) and the corresponding peptides were eluted from the gel using trifluoroacetic acid. Mass spectrometry analysis and protein identification was done as described previously [1]

**Culture Supernatant preparation and FACS-based erythrocyte binding assay (EBA)**

*P. falciparum* culture supernatant of schizont infected erythrocytes was prepared as described previously [2, 3] and was used as a source of soluble parasite proteins. Briefly, erythrocytes were incubated with 0.5 ml of culture supernatant at 37°C for 3 hours. After incubation, the erythrocytes were washed with PBS and bound proteins were detected using specific antibodies and respective FITC conjugated antibodies and were read on a FACS Caliber flow cytometer. The resulting flow cytometry data were analyzed using Cell Quest software.

**Co-Immunoprecipitation and Mass Spectrometric Analysis**

Immunoprecipitation experiments using lysate of the schizont stage parasites were performed as prescribed (Thermo scientific). The trypsin digested samples were analyzed on a nano-LC equipped Orbitrap VELOS PRO (Thermo Fisher Scientific) mass spectrometer as described previously [1]. The proteins were identified by blasting the peptides over a *Plasmodium falciparum* database (Uniprot), using proteome discoverer (Thermofisher) using standardized procedure as described earlier [1].

**Invasion Inhibition Assay**

Invasion inhibition assays to determine the parasite neutralizing ability of PfARO antibodies were conducted as described earlier [3, 4]. Purified anti-PfARO rabbit IgGs were tested at different concentrations for their ability to block erythrocyte invasion of *P. falciparum* 3D7 parasites. Briefly, schizont stage parasites were incubated (0.3% parasitemia; 2% hematocrit) for 40 hours with different concentrations of IgG’s purified from pre-immune or immune sera. The newly infected erythrocytes were labelled by ethidium bromide and scored using flow cytometry. Invasion inhibition for immune IgG was estimated with respect to the control pre-immune IgG from the same animal.

**Legends**

**Figure S1: Specificity of antibodies raised against recombinant rPfARO.**

**A.** Immunoblot analysis performed using anti-PfARO mice and rabbit antibodies detected rPfARO whereas the corresponding pre-immune control antibodies failed to recognize rPfARO.

**B.** Immunofluorescence analysis of late schizont stage parasites show that the mice pre-immune antibodies failed to produce any fluorescent signal suggesting that they did not non-specifically detect or cross-react with any other parasite protein. Whereas, in the same parasite section, the PfRH2 antibodies exhibited an apical staining consistent with its localization to the rhoptry.

**Figure S2: PfARO does not exhibit erythrocyte binding activity.**

**A.** Native PfARO present in the parasite culture supernatant does not show any erythrocyte binding activity. **B.** Native PfEBA-175 was analyzed as a positive control from the same parasite supernatant preparation and was observed to exhibit potent erythrocyte binding activity.

**Figure S3.** **PfARO antibodies do not exhibit invasion inhibitory activity.**

Total rabbit IgGs against PfARO were evaluated for their invasion inhibitory activity against the *P. falciparum* strain 3D7. PfARO purified total IgG did not exhibit any significant invasion inhibition, while as a positive control the MSP-Fu purified total IgGs exhibited a potent invasion inhibition [5].

**References**

1. Reddy KS, Amlabu E, Pandey AK, Mitra P, Chauhan VS, Gaur D. Multiprotein complex between the GPI-anchored CyRPA with PfRH5 and PfRipr is crucial for *Plasmodium falciparum* erythrocyte invasion. Proc Natl Acad Sci U S A. 2015; 112(4):1179-84.
2. Gaur D, Singh S, Jiang L, Diouf A, Miller LH. Recombinant *Plasmodium falciparum* reticulocyte homology protein 4 binds to erythrocytes and blocks invasion. Proc Natl Acad Sci U S A. 2007; 104: 17789-17794.
3. Sahar T, Reddy KS, Bharadwaj M, Pandey AK, Singh S, Chitnis CE, et al. *Plasmodium falciparum* reticulocyte binding-like homologue protein 2 (PfRH2) is a key adhesive molecule involved in erythrocyte invasion. PloS One. 2011; 6: e17102.
4. Reddy KS, Pandey AK, Singh H, Sahar T, Emmanuel A, Chitnis CE, et al. Bacterially expressed full-length recombinant *Plasmodium falciparum* RH5 protein binds erythrocytes and elicits potent strain-transcending parasite-neutralizing antibodies. Infect Immun. 2014; 82:152-164.
5. Gupta PK, Mukherjee P, Dhawan S*,* Pandey AK, Mazumdar S, Gaur D, et al. Production and preclinical evaluation of *Plasmodium falciparum* MSP-1_19_ and MSP-3_11_ chimeric protein, PfMSP-Fu_24_. Clin Vaccine Immunol. 2014; 21: 886-897.
